# Population‐based, nationwide registration of prostatectomies in Sweden

**DOI:** 10.1002/jso.25643

**Published:** 2019-07-29

**Authors:** Walter Cazzaniga, Rebecka Arnsrud Godtman, Stefan Carlsson, Göran Ahlgren, Eva Johansson, David Robinson, Jonas Hugosson, Pär Stattin

**Affiliations:** ^1^ Division of Experimental Oncology/Unit of Urology URI, IRCCS Ospedale San Raffaele University Vita‐Salute San Raffaele Milan Italy; ^2^ Department of Surgical Sciences Uppsala University Uppsala Sweden; ^3^ Department of Urology, Institute of Clinical Sciences Sahlgrenska Academy at University of Göteborg, Sahlgrenska University Hospital Göteborg Sweden; ^4^ Division of Urology Karolinska University Hospital Stockholm Sweden; ^5^ Department of Molecular Medicine and Surgery (MMK) Karolinska Institutet Stockholm Sweden; ^6^ Department of Urology, Lund University Skåne University Hospital Skåne Sweden; ^7^ Department of Urology Ryhov Hospital Jonköping Sweden

**Keywords:** NPCR, prostate cancer, radical prostatectomy, registry

## Abstract

**Introduction:**

Radical prostatectomy (RP) is a common surgical procedure with a risk of postoperative erectile dysfunction and urinary incontinence. There is a need for data on RP as a basis for quality assurance and benchmarking.

**Methods:**

In 2015, prostatectomies in Sweden (PiS) form was implemented in the National Prostate Cancer Register (NPCR) of Sweden with data on pre‐, peri‐ and post‐operative variables.

**Results:**

Out of all radical prostatectomies performed in 2016 in Sweden, 3096/3881 (80%) were registered in PiS. A total of 2605 (84%) were robot‐assisted radical prostatectomy (RARP) and 491 (16%) were RRP (retropubic radical prostatectomy). RARP was performed by 91 surgeons of whom 47% operated more than 25 RP/year; and RRP was performed by 69 surgeons of whom 10% performed more than 25 RP/year. RARP had a longer operative time (median operating time: RARP 155 minutes [IQR 124‐190]; RRP 129 minutes [IQR 105‐171]; *P* < .001) but was associated with smaller bleeding (median intraoperative blood loss: RARP 100 mL [IQR 50‐200], RRP 700 mL [IQR 500‐1100]; *P* < .001).

**Conclusions:**

We report on a nationwide, population‐based register with transparent reporting of data on the performance of radical prostatectomy. These data are needed as a basis for quality assurance with comparisons of results from individual surgeons and hospitals.

## 1 INTRODUCTION

Radical prostatectomy (RP) is a common surgical procedure used worldwide as curative treatment for prostate cancer (Pca). However, there is a substantial risk of erectile dysfunction and urinary incontinence postoperatively.^1^, ^2^, ^3^ High surgical volume has been associated with better outcomes including better cancer control and less postoperative erectile dysfunction and urinary incontinence. Furthermore, there is a large variation between individual surgeons, also among those who perform a large number of RP's.^4^, ^5^ Therefore, there is a need for a uniform registration of data on pre‐, peri, and post‐operative variables after RP including case mix as a basis for quality assurance and benchmarking of individual surgeons and hospitals.

In 2015, a form for prostatectomies in Sweden (PiS) was implemented in the National Prostate Cancer Register of Sweden.^6^ The aim of this form is to collect comprehensive data for men with Pca who undergo RP to support quality assurance and quality improvement. There are no legal obligations for a department to perform this registration. For research purposes, there is also a need for more data on the cases such as, for example, socioeconomic status, comorbidity to assess case mix but to minimize the data collection in PiS, these data are captured by cross linkages to other nationwide population‐based health care registries and demographic databases. Here, we report the preliminary results obtained in PiS.

## 2 MATERIALS AND METHODS

### 2.1 The National Prostate Cancer Register (NPCR) of Sweden

The National Prostate Cancer Register of Sweden captures comprehensive data for 98% of all incident cases of Pca in Sweden with the aim to assess health care for men with Pca.^6^

Data in NPCR are collected by the use of four forms: a diagnostic form with information on diagnostic characteristics, primary treatment and work‐up form with information on subsequent work‐up and medical treatment and two separate treatment forms for curatively intended procedures, one for radiotherapy and one for prostatectomy (PiS). The aim of this paper was to describe the content of the PiS form.

### 2.2 PiS form

Two versions of a PiS form has been in use since January 2015, a shorter version with 60 variables and an extensive version with 83 variables. The version that is used is determined by each reporting department.

Table 1 reports the complete list of variables and the capture for each variable which was defined as the percentage of nonmissing values out of the total number of cases recorded for each variable for the short and extensive form respectively, in 2016.

**Table 1:** Capture of variables in the prostatectomy in Sweden (PiS) form in the National Prostate Cancer Register (NPCR)

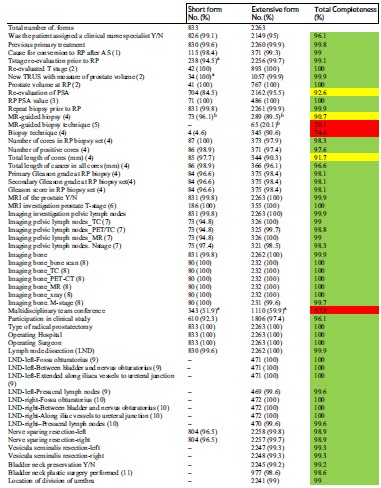
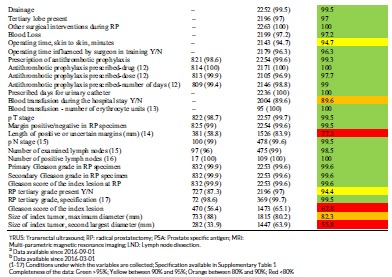

Overall, out of 3096 procedures registered, 833 (27%) were recorded by use of the short form and 2263 (73%) with the extensive form.

The collected variables include information regarding the preoperative characteristics, for example, data on the histopathological characteristics in the core biopsies, data on TNM classification, results from imaging, perioperative data, for example, type of RP, operative time, perioperative blood loss, hospital and surgeon code, and postoperative data, for example, histopathological assessment of surgical specimen, prescribed drug therapy after surgery etc.

A full variable list for the two PiS forms is available in Swedish at: https://www.cancercentrum.se/globalassets/cancerdiagnoser/prostatacancer/dokument/radikalop_manual_2018.pdf

Specifically, 70/83 (84%) variables had ≥95% of completeness, 5/83 (6%) had a completeness between 90% and 95%, 2/83 (2%) had a total completeness between 80% and 90% and 6/83 (7%) had a completeness below 80%. Specification regarding the inclusion criteria for some specific variable is reported in Table S1.

**Figure 1:**
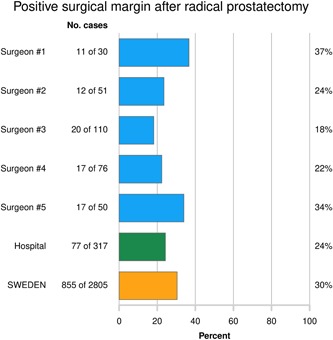
Display of proportion of positive margin per surgeon at a department, the mean for all surgeons at the hospital, and the mean for all surgeons in the nation. Data for a department are displayed 24 hours after reporting at the secured server Information Network for Cancer Care for this department, several other variables besides positive margins including operating time and preoperative blood loss are also reported in a similar fashion. The demonstrated data are fictitious [Color figure can be viewed at wileyonlinelibrary.com]

These data are subsequently reported online at the secured Information Network for Cancer Care (INCA) platform within 24 hours to the reporting unit with comparisons between surgeons at the department, and the average for the health care region as well as for the entire nation (Figure 1).^7^ In addition, the number of RP's performed per year at each hospital and the number of RP's per surgeon is publicly reported at www.npcr.se/RATTEN in April for the preceding year.^8^

NPCR has been linked with other national healthcare registries and demographic databases to obtain information on comorbidities, socioeconomic factors, and outcome in Prostate Cancer data Base Sweden (PCBaSe).^9^, ^10^

The Longitudinal Integration Database for Health Insurance and Labor Market Studies (LISA)^11^ holds information regarding educational level, income, civil status, and type of employment.

The National Patient Registry^12^ holds information regarding all in‐patient care in Sweden from 1987 including surgical procedures and dates of admission and discharge. The Charlson comorbidity index (CCI) was calculated by the use of discharge diagnoses in this registry based on data up to 10 years before the date of the RP, as previously described.^9^

## 3 RESULTS

**Figure 2:**
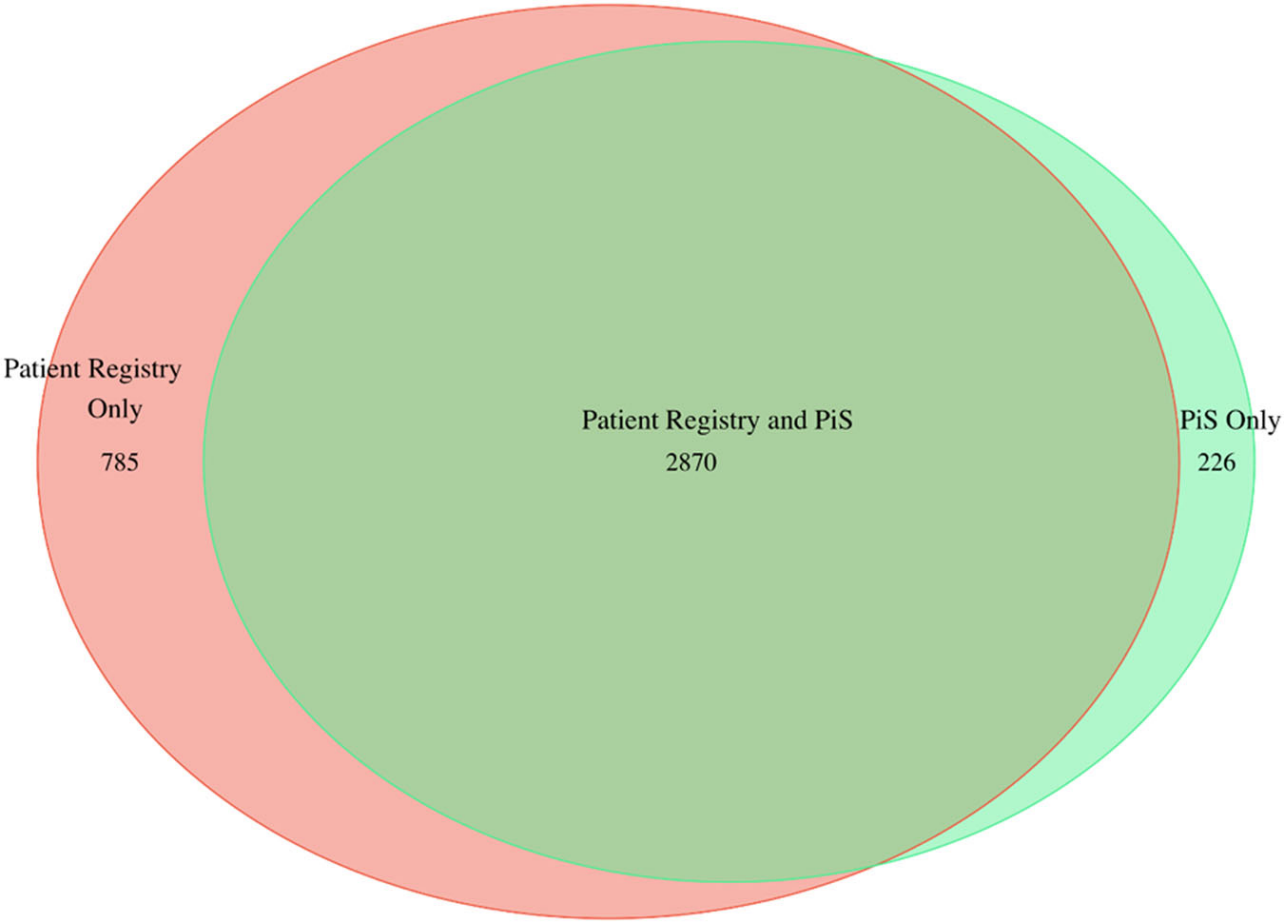
Number of radical prostatectomies registered in The Patient Registry only, Patient Registry and in the prostatectomies in Sweden (PiS) form, and in PiS only in 2016 [Color figure can be viewed at wileyonlinelibrary.com]

Out of all 3881 RP's performed and registered in Sweden in 2016, 3096 (80%) RP's had been registered with a PiS form, 2870 (92%) had also been reported to the National Patient Registry, while 226 (8%) had been reported with a PiS form only and 785 had been registered in the Patient Registry only (Figure 2). To assess the characteristics of men for whom RP was registered in one of the two registers or in both registers, we compared data in NCR (except for the data retrieved in PiS) for men who were registered with a PiS form with those who had their RP registered only the Patient Registry (Table S2).

Men reported with a PiS form only were more frequently operated in the metropolitan areas of Västra Götaland and Stockholm where a high proportion of RP's are performed in private clinics, and these men were younger and had more often undergone a robot‐assisted radical prostatectomy (RARP).

Overall, 2440 (79%) of the prostatectomies were performed as primary treatment and 570 (18%) after an initial period of active surveillance.

A total of 2605 (84%) RP's were performed as RARP and 491 (16%) were performed as retropubic radical prostatectomy (RRP) (Table 2). Median age at date of RP was 65 years (IQR 60‐69) for RARP and 67 years (IQR 62‐70) for RRP. Men who underwent RARP had a lower number of cores per diagnostic biopsy session compared with the RRP group (median number of biopsies: RARP 10 [IQR 10‐12]; RRP 12 [IQR 10‐12]; *P* < .001). The number of biopsy procedures from the first diagnosis date to the radical prostatectomy date is not collected in PiS form. However, possible explanations for difference in the number of biopsy cores are that magnetic resonance imaging (MRI) guided biopsies were more commonly used in the first diagnostic set before RARP than before RRP (12% vs 7%). Despite the similar proportion of re‐biopsies before RP (RARP 16% vs RRP 13%), the use of biopsies guided by MRI was three times higher before RARP than before RRP (RARP 24% vs RRP 8%).

**Table 2:** Preoperative characteristics of men who underwent radical prostatectomy in Sweden and registered in prostatectomy in Sweden (PiS) form in 2016

	RARP	RRP
No. procedures (%)	2605 (84.1)	491 (15.9)
Age, y		
Median (IQR)	65.0 (60.0‐69.0)	67.0 (62.0‐70.0)
PSA (ng/mL)		
Median (IQR)	6.6 (4.5‐10.0)	7.5 (5.2‐11.4)
PSA	No (%)	
<3 ng/mL	99 (3.8)	6 (1.2)
3 to 10 ng/mL	1863 (71.5)	337 (68.6)
10.1 to 20 ng/mL	462 (17.7)	97 (19.8)
>20 ng/mL	175 (6.7)	48 (9.8)
Missing	6 (0.2)	3 (0.6)
Prostate volume		
Median (IQR)	35 (28‐47)	38 (30.50)
<30 gr	757 (29.1)	111 (22.6)
30 to 60 gr	1441 (55.3)	282 (57.4)
60 to 90 gr	251 (9.6)	55 (11.2)
>90 gr	65 (2.5)	14 (2.9)
Missing	91 (3.5)	29 (5.9)
Number of cores		
Median (IQR)	10 (10‐12)	12 (10‐12)
≤9	232 (8.9)	48 (9.8)
10 to 12	2187 (84)	401 (81.7)
≥13	146 (5.6)	33 (6.7)
Missing	40 (1.5)	9 (1.8)
Number of positive cores		
Median (IQR)	4 (2–6)	4 (2–6)
≤2	832 (31.9)	136 (27.7)
3 to 4	783 (30.1)	136 (27.7)
5 to 6	543 (20.8)	105 (21.4)
>6	402 (15.4)	103 (21.0)
Missing	45 (1.7)	11 (2.2)
Total mm of cancer in cores		
Median (IQR)	11.5 (4.8‐23.0)	13.2 (5.9‐31.0)
cT Stage		
T1a/T1b	29 (1.1)	10 (2.0)
T1c	1647 (63.2)	289 (58.9)
T2	797 (30.6)	174 (35.4)
T3	89 (3.4)	14 (2.9)
T4	1 (0.0)	1 (0.2)
Missing	42 (1.6)	3 (0.6)
cN Stage		
N0	1044 (40.1)	190 (38.7)
N1	41 (1.6)	10 (2.0)
NX	1520 (58.3)	291 (59.3)
Missing	0 (0.0)	0 (0.0)
cM Stage		
M0	2546 (97.7)	484 (98.6)
M1	10 (0.4)	3 (0.6)
MX	49 (1.9)	4 (0.8)
Missing	40 (0.0)	0 (0.0)
Gleason grade groups		
GGG1	905 (34.7)	138 (28.1)
GGG2	1088 (41.8)	186 (37.9)
GGG3	341 (13.1)	94 (19.1)
GGG4	174 (6.7)	39 (7.9)
GGG5	82 (3.1)	32 (6.5)
Missing	15 (0.6)	2 (0.4)
Risk category		
Very low‐risk	131 (5.0)	28 (5.7)
Low‐risk	552 (21.2)	64 (13.0)
Intermediate‐risk	1388 (53.3)	278 (56.6)
High‐risk	320 (12.3)	84 (17.1)
Locally advanced	79 (3.0)	9 (1.8)
Regionally metastatic	62 (2.4)	16 (3.3)
Distant metastasis	15 (0.6)	4 (0.8)
Missing	58 (2.2)	8 (1.6)
Charlson Comorbidity Index		
0	2246 (86.2)	398 (81.1)
1	249 (9.6)	56 (11.4)
2	76 (2.9)	30 (6.1)
3+	34 (1.3)	7 (1.4)
Missing	0 (0.0)	0 (0.0)
Educational level		
Low	519 (19.9)	141 (28.7)
Intermediate	1149 (44.1)	228 (46.4)
High	921 (35.4)	121 (24.6)
Missing	16 (0.6)	1 (0.2)
Civil status		
Unmarried	807 (31.0)	179 (36.5)
Married	1795 (68.9)	312 (63.5)
Missing	3 (0.1)	0 (0.0)
Income		
Q1	616 (23.6)	156 (31.8)
Q2	645 (24.8)	125 (25.5)
Q3	650 (25.0)	135 (27.5)
Q4	691 (26.5)	75 (15.3)
Missing	3 (0.1)	0 (0.0)

*Note*: Number of cores = number of cores in biopsy session in which Pca was diagnosed. The National Care Program recommends that 10 to 12 cores should be obtained in the first set of systematic biopsies; Educational level: low = compulsory school, <10 years; intermediate = upper secondary school, 10–12 years; high = college or university, >12 years; Quartile of income: Q1 lowest – Q4 highest; Risk categories: very low‐risk (T1c, GGG1 [GS 6], Prostate‐specific antigen [PSA] <10 ng/mL, PSA density <0.15, number of cores positive for cancer ≤4, cancer extension at biopsy <8 mm), low‐risk (T1‐2, PSA <10 ng/mL and GGG1), intermediate‐risk (T1‐2, GGG2 or 3 [GS 7] and/or PSA 10 to <20 ng/mL), high‐risk (T3 and/or GGG 4 or 5 GS 8‐10 and/or PSA 20 to 50 ng/mL), very high‐risk (T4, PSA 50 to 200 ng/mL, any N stage, M0), regionally metastatic (T4 and/or N1 and/or PSA 50 to 100 ng/mL in the absence of distant metastases [M0 or Mx]), and distant metastases (PSA above 100 ng/mL or M1).

Abbreviations: IQR, inter‐quartile range; PSA, prostate‐specific antigen; RARP, robot‐assisted radical prostatectomy; RRP, retropubic radical prostatectomy.

Irrespective of type of RP, the majority of men were diagnosed with an intermediate‐risk Pca (53% RARP vs 56% RRP) and had a CCI of zero.

Men who underwent RARP had a slightly longer operative time (median operating time: RARP 155 minutes [IQR 124‐190]; RRP 129 minutes [IQR 105‐171]; *P* < .001) but smaller blood loss (median intraoperative blood loss: RARP 100 mL [IQR 50–200], RRP 700 mL [IQR 500‐1100]; *P* < .001) (Table 3). Furthermore, men who underwent RARP more frequently received a nerve sparing procedure compared to men who underwent a RRP (RARP 79% vs RRP 50%), more frequently underwent a lymph node dissection (RARP 16% vs RRP 12%), more often had stage pT2 (RARP 61% vs RRP 55%) but less often had pT3 (RARP 8% vs RRP 15%) and were upstaged less often (RARP 27% vs RRP 35%).

**Table 3:** Peri‐ and post‐operative performance data on radical prostatectomy in 2016

	RARP	RRP
	No (%)	
Operation time, min		
Median (IQR)	155 (124‐190)	129.5 (105‐171)
≤120	435 (16.7)	117 (23.8)
120 to 150	453 (17.4)	62 (12.6)
150 to 180	438 (16.8)	36 (7.3)
>180	545 (20.9)	57 (11.6)
Missing	734 (28.2)	219 (44.6)
Blood loss, mL		
Median (IQR)	100 (50‐200)	700 (500‐1100)
<100	619 (23.8)	1 (0.2)
100 to 249	951 (36.5)	11 (2.2)
250 to 499	273 (10.5)	44 (9.0)
500 to 999	75 (2.9)	120 (24.4)
≥1000	12 (0.5)	93 (18.9)
Missing	675 (25.9)	222 (45.2)
Lymph node dissection		
Not performed	2098 (80.5)	417 (84.9)
Limited	14 (0.5)	6 (1.2)
Extended	404 (15.5)	52 (10.6)
Missing	89 (3.4)	16 (3.3)
Nerve sparing procedure		
Yes	2062 (79.2)	246 (50.1)
No	539 (20.7)	220 (44.8)
Missing	4 (0.2)	25 (5.1)
Surgical margin status		
Negative	1731 (66.8)	324 (66.4)
Positive	790 (30.5)	134 (27.5)
Missing	70 (2.7)	30 (6.1)
pT stage
pT0	8 (0.3)	1 (0.2)
pT2	1586 (60.9)	271 (55.2)
pT3a	776 (29.8)	138 (28.1)
pT3b	216 (8.3)	73 (14.9)
pT4	8 (0.3)	2 (0.4)
Missing	11 (0.4)	6 (1.2)
pN stage		
N0	399 (78.7)	53 (71.6)
N1	106 (20.9)	20 (27.0)
Missing	2 (0.4)	1 (1.4)
Upgrading		
No	1596 (61.3)	321 (65.4)
Yes	987 (37.9)	166 (33.8)
Missing	22 (0.8)	4 (0.8)
Upstaging		
No	1810 (69.5)	294 (61.9)
Yes	727 (27.9)	172 (35.2)
Missing	68 (2.6)	25 (2.9)

*Note*: Limited lymph node dissection (LND): LND performed at the level of the obturator fossa including the obturatory nerve area; Extended LND: extended to the presacral region; Upgrading: defined as a GGG (Gleason Grade Group) at pathological specimen of radical prostatectomy higher than the GGG in biopsies; Upstaging: tumor diagnosed as a T1a/b, T1c or T2 preoperatively that was found to be a pT3a, pT3b or pT4 at examination of RP specimen or a T3 tumor at diagnosis is found to be a pT4 or N0 preoperatively found that had N1 disease at examination of RP specimen.

Abbreviations: RARP, robot‐assisted radical prostatectomy; RRP, retropubic radical prostatectomy.

**Figure 3:**
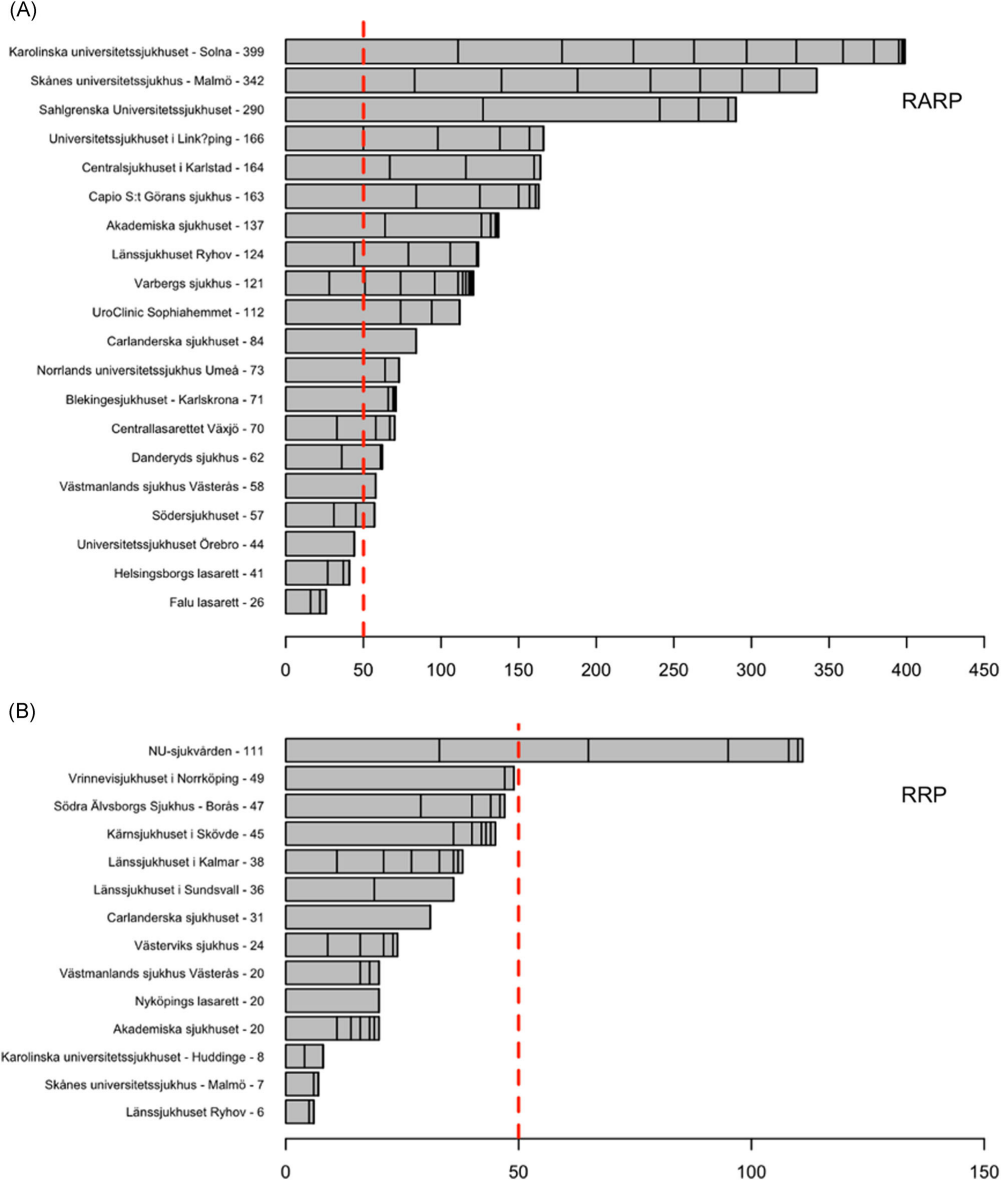
Number of radical prostatectomies recorded in prostatectomy in Sweden for each hospital in 2016. Area between vertical lines represents the number of RP's for each single surgeon. Red dashed line represents limit for low volume (50 RP's/year) as defined in the Swedish National Prostate Cancer Care programme (at least two surgeons that each perform 25 or more RP's). Departments where less than 5 RP's are not displayed. RP, radical prostatectomy [Color figure can be viewed at wileyonlinelibrary.com]

Data were reported from 20 departments performing RARP and 14 departments performing RRP. At 17/20 departments (85%) where RARP was used, more than 50 procedures were performed whereas, for RRP, only one out of 14 departments performed more than 50 procedures (Figure 3). The Swedish national guidelines for prostate cancer care recommends that each RP surgeon should perform 25 or more RP's per year and that there should be at least two RP surgeons at each department.

Overall, 76% of all RPs were performed by surgeons who performed more than 25 RP/year.

A total of 91 surgeons were registered performing RARP and 69 performing RRP; 47% of RARP surgeons performed more than 25 RP/year while only 10% of RRP surgeons performed 25 procedures or more.

**Figure 4:**
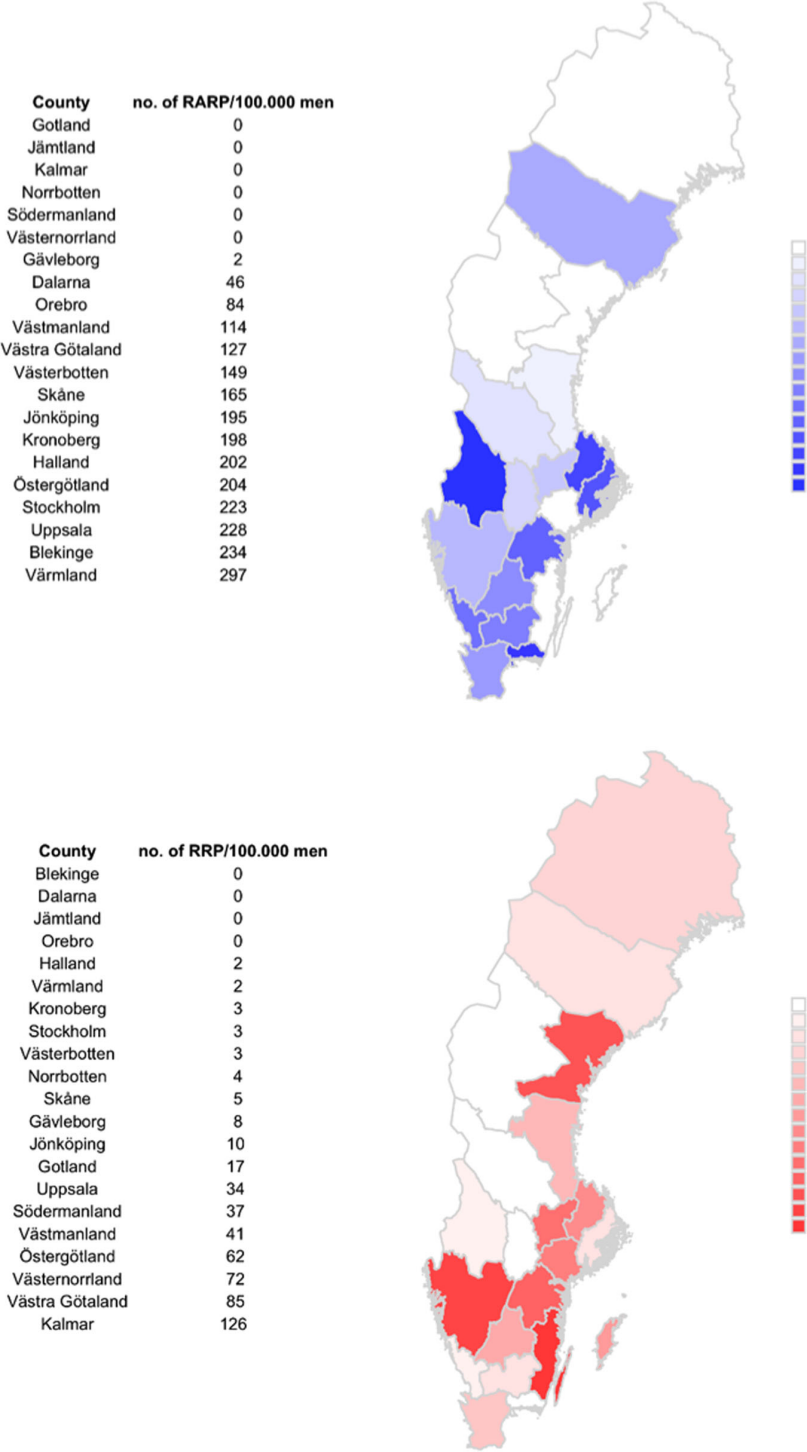
Age‐standardized incidence of prostatectomies per county in Sweden in 2016. No data were delivered from the county of Jämtland regarding RP performed in 2016 to NPCR. In the Jämtland county the incidence of RRP and RRP combined was 157/100.000 according to the Patient Registry. RP, Radical prostatectomy; RRP, retropubic radical prostatectomy [Color figure can be viewed at wileyonlinelibrary.com]

There were large variations in the use of RP among the Swedish counties (Figure 4). The highest number of RARP per 100 000 men in Värmland county (297/100 000 men) and the highest number of RRP in Kalmar county (126/100 000 men).

## 4 DISCUSSION

The introduction of a national registration of radical prostatectomy in a clinical cancer register shows that such a registration is feasible even without being mandated by law.

Limitations of our data collected by use of a specific form for prostatectomy in NPCR are that capture was not complete and compared to compulsory registration to the Patient Registry there was some selection of younger and healthier men in our registration as these men more often underwent RARP in private practise. However, a capture rate of 80% already in the second year of the registration and a high completeness data for each variable in the PiS form are strengths of our registration.

Other initiatives in the same area include the British Association of Urological Surgeons (BAUS) RP audit with a complete capture of all RP's performed in England with pre‐, peri and post‐operative data per surgeon (https://www.baus.org.uk/_userfiles/pages/files/Publications/Audit/Radical_Prostatectomy_2016_final_analysis.pdf).

We believe these registers will become useful tools for quality assurance and benchmarking. Rapid feedback to health care providers is necessary and public, transparent reporting is necessary to achieve the optimal impact of such data.^8^

## 5 CONCLUSIONS

It is possible to collect detailed data on cancer characteristics, case mix, case load, surgical method, and technical aspects of the performance of radical prostatectomy in a nationwide, population‐based register. Rapidly and transparently reported, these data are useful tools for quality assurance.

## CONFLICT OF INTERESTS

The authors declare that there are no conflict of interests.

## AUTHOR CONTRIBUTIONS

JH, PS, SC, GA, EJ, and DR created and implemented the PiS form and contributed with data. WC and PS analyzed data and wrote the manuscript. All authors contributed to the final manuscript and approved the final version to be published.

## DATA SHARING STATEMENT

Researchers can apply for collaborations based on data in PCBaSe including PiS with a standardized form. After approval, a study file will be uploaded to a remote access server for statistical analysis. Users will be charged for software licenses, administration, and data management. For more information, contact: npcr@npcr.se.

## SYNOPSIS

We report on a nationwide, population‐based register with transparent reporting of data on the performance of radical prostatectomy. These data are useful tools for quality assurance.

## Supporting information

Supporting information
