# Supplementary material for: Population‐based, nationwide registration of prostatectomies in Sweden
Source: J Surg Oncol. 2019 Jul 29;120(4):803–12. doi: 10.1002/jso.25643 (PMC6771627; doi:10.1002/jso.25643)
Supplement: Supplementary file 1 — Supporting information [file JSO-120-803-s001.docx]

# SUPPLEMENTARY DATA

**Supplementary Table 1.** Specification of variables registered in PiS

| (1) Available only for men with a previous primary treatment strategy (92% after active surveillance) |
| --- |
| (2) Available only for men who had been re-evaluated after the initial diagnosis |
| (3) Available only for men who had undergone a new PSA test after diagnosis |
| (4) Available only for men who had undergone a new biopsy after the initial diagnosis |
| (5) Available only for men who had undergone a new MR-guided biopsy after the initial diagnosis |
| (6) Available only for men who had undergone an MRI before radical prostatectomy |
| (7) Available only for men who had and so on been investigated with an imaging technique for lymph node invasion |
| (8) Available only for men who have been investigated with an imaging technique for bone metastasis |
| (9) Available only for men who have been submitted to lymph node dissection (bilateral or left sided) |
| (10) Available only for men who have been submitted to lymph node dissection (bilateral or right sided) |
| (11) Available only for men who haven’t been submitted to bladder neck dissection |
| (12) Available only for men who have had an antithrombotic drug prescribed |
| (13) Available only for men who received a blood transfusion |
| (14) Available only for men with positive or uncertain surgical margins |
| (15) Available only for men submitted to lymph node dissection (LND) |
| (16) Available only for men submitted to lymph node dissection due to N1 disease |
| (17) Available only for men with a tertiary Gleason Grade at RP |

**Supplementary Table 2.** Characteristics for men registered with RP in PiS form only, PiS and Patient Registry, and Patient Registry only

|  | PiS only | PiS and Patient Registry | Patient Registry only** |
| --- | --- | --- | --- |
| No. of procedure | 226 | 2 870 | 394 |
| Age (yr) |  |  |  |
| Median (IQR) | 63.0 (58.0-68.0) | 66.0 (60.0-69.0) | 68.0 (63.0-72.0) |
| PSA no.(%) |  |  |  |
| <3 ng/ml | 12 (5.3) | 93 (3.2) | 21 (5.3) |
| 3-10 ng/ml | 160 (70.8) | 2040 (71.1) | 193 (49.0) |
| 10.1-20 ng/ml | 44 (19.5) | 515 (17.9) | 56 (14.2) |
| >20 ng/ml | 8 (3.5) | 215 (7.5) | 22 (5.6) |
| Missing | 2 (0.9) | 7 (0.2) | 102 (25.9) |
| Risk category no.(%) |  |  |  |
| Very low risk | 12 (5.3) | 147 (5.1) | 24 (6.1) |
| Low risk | 52 (23.0) | 564 (19.7) | 69 (17.5) |
| Intermediate risk | 122 (54.0) | 1544 (53.8) | 115 (29.2) |
| High risk | 32 (14.2) | 372 (13.0) | 36 (9.1) |
| Locally advanced | 1 (0.4) | 87 (3.0) | 16 (4.1) |
| Regionally metastatic | 5 (2.2) | 73 (2.5) | 10 (2.5) |
| Distant Metastasis | 0 (0.0) | 19 (0.7) | 10 (2.5) |
| Missing | 2 (0.9) | 64 (2.2) | 114 (28.9) |
| Primary vs Secondary no.(%) |  |  |  |
| Primary | 188 (83.2) | 2252 (78.5) | 38 (9.6) |
| Secondary | 35 (15.5) | 567 (19.8) | 110 (27.9) |
| Missing | 3 (1.3) | 51 (1.8) | 246 (62.4) |
| RP type no.(%) |  |  |  |
| RRP | 44 (19.5) | 447 (15.6) | 262 (66.5) |
| RARP | 182 (80.5) | 2423 (84.4) | 132 (33.5) |
| Missing | 0 (0.0) | 0 (0.0) | 0 (0.0) |
| Counties no.(%) |  |  |  |
| Blekinge | 22 (9.7) | 49 (1.7) | 1 (0.3) |
| Dalarna | 0 (0.0) | 26 (0.9) | 0 (0.0) |
| Gävleborg | 0 (0.0) | 5 (0.2) | 0 (0.0) |
| Gotland | 0 (0.0) | 2 (0.1) | 1 (0.3) |
| Halland | 1 (0.4) | 121 (4.2) | 0 (0.0) |
| Jonkoping | 1 (0.4) | 129 (4.5) | 1 (0.3) |
| Kalmar | 0 (0.0) | 62 (2.2) | 1 (0.3) |
| Kronoberg | 0 (0.0) | 71 (2.5) | 0 (0.0) |
| Norrbotten | 0 (0.0) | 2 (0.1) | 0 (0.0) |
| Orebro | 0 (0.0) | 44 (1.5) | 0 (0.0) |
| Östergötland | 3 (1.3) | 212 (7.4) | 3 (0.8) |
| Skåne | 7 (3.1) | 385 (13.4) | 12 (3.0) |
| Södermanland | 0 (0.0) | 21 (0.7) | 45 (11.4) |
| Stockholm | 65 (28.8) | 739 (25.7) | 24 (6.1) |
| Uppsala | 0 (0.0) | 157 (5.5) | 0 (0.0) |
| Värmland | 1 (0.4) | 164 (5.7) | 1 (0.3) |
| Västerbotten | 0 (0.0) | 74 (2.6) | 0 (0.0) |
| Västernorrland | 0 (0.0) | 36 (1.3) | 0 (0.0) |
| Västmanland | 0 (0.0) | 78 (2.7) | 0 (0.0) |
| Västra Götaland | 126 (55.8) | 493 (17.2) | 2 (0.5) |
| Missing | 0 (0.0) | 0 (0.0) | 303 (76.9) |
| CCI no.(%) |  |  |  |
| 0 | 203 (89.8) | 2441 (85.1) | 243 (61.7) |
| 1 | 18 (8.0) | 287 (10.0) | 24 (6.1) |
| 2 | 3 (1.3) | 103 (3.6) | 81 (20.6) |
| 3+ | 2 (0.9) | 39 (1.4) | 46 (11.7) |
| Educational level no.(%) |  |  |  |
| Low | 24 (10.6) | 636 (22.2) | 107 (27.2) |
| Intermediate | 75 (33.2) | 1302 (45.4) | 178 (45.2) |
| High | 124 (54.9) | 918 (32.0) | 107 (27.2) |
| Missing | 3 (1.3) | 14 (0.5) | 2 (0.5) |
| Income no.(%) |  |  |  |
| Q1 | 27 (11.9) | 745 (26.0) | 144 (36.5) |
| Q2 | 23 (10.2) | 747 (26.0) | 106 (26.9) |
| Q3 | 62 (27.4) | 723 (25.2) | 71 (18.0) |
| Q4 | 113 (50.0) | 653 (22.8) | 73 (18.5) |
| Missing | 1 (0.4) | 2 (0.1) | 0 (0.0) |
| Hospital Type no.(%) |  |  |  |
| Private | 178 (79.2) | 211 (7.4) | 22 (5.6) |
| Public | 47 (20.8) | 2659 (92.6) | 372 (94.4) |

RRP: Retropubic radical prostatectomy; RARP: Robot-assisted radical prostatectomy; IQR: Inter-quartile range; PSA: Prostate specific antigen; Educational level: low = compulsory school, <10 years; intermediate = upper secondary school, 10–12 years; high = college or university, >12 years; Quartile of income: Q1 lowest – Q4 highest; Risk categories: very low‐risk (T1c, GGG1 (GS 6), Prostate‐specific antigen (PSA) <10 ng/ml, PSA density < 0.15, number of cores positive for cancer ≤4, cancer extension at biopsy < 8mm), low‐risk (T1‐2, PSA <10 ng/mL and GGG1), intermediate‐risk (T1‐2, GGG2 or 3 (GS 7) and/or PSA 10 to <20 ng/ml), high‐risk (T3 and/or GGG 4 or 5 GS 8-10) and/or PSA 20 to 50 ng/ml), very high-risk (T4, PSA 50 to 200 ng/ml, any N stage, M0), regionally metastatic (T4 and/or N1 and/or PSA 50 to 100 ng/ml in the absence of distant metastases (M0 or Mx), and distant metastases (PSA above 100 ng/ml or M1); Data for the “Patient Registry only” group were retrieved from the diagnostic and work-up forms in NPCR.

** 394 out of 785 men (50%) who had undergone RP according to the Patient Registry had been registered in NPCR by use of the diagnostic and workup forms
